# Central GLP-1 receptor signalling accelerates plasma clearance of triacylglycerol and glucose by activating brown adipose tissue in mice

**DOI:** 10.1007/s00125-015-3727-0

**Published:** 2015-08-09

**Authors:** Sander Kooijman, Yanan Wang, Edwin T. Parlevliet, Mariëtte R. Boon, David Edelschaap, Gido Snaterse, Hanno Pijl, Johannes A. Romijn, Patrick C. N. Rensen

**Affiliations:** Department of Medicine, Division of Endocrinology, Leiden University Medical Center, Room C7-Q44, Albinusdreef 2, PO Box 9600, 2300 RC Leiden, the Netherlands; Einthoven Laboratory for Experimental Vascular Medicine, Leiden University Medical Center, Leiden, the Netherlands; Department of Medicine, Academic Medical Center, Amsterdam, the Netherlands

**Keywords:** Brown adipose tissue, Exendin-4, GLP-1, Mice, Triacylglycerol

## Abstract

**Aims/hypothesis:**

Glucagon-like peptide 1 (GLP-1) receptor (GLP-1R) agonism, used in the treatment of type 2 diabetes, has recently been shown to increase thermogenesis via the brain. As brown adipose tissue (BAT) produces heat by burning triacylglycerol (TG) and takes up glucose for de novo lipogenesis, the aim of this study was to evaluate the potential of chronic central GLP-1R activation by exendin-4 to facilitate clearance of lipids and glucose from the circulation by activating BAT.

**Methods:**

Lean and diet-induced obese (DIO) C57Bl/6J mice were used to explore the effect of a 5 day intracerebroventricular infusion of the GLP-1 analogue exendin-4 or vehicle on lipid and glucose uptake by BAT in both insulin-sensitive and insulin-resistant conditions.

**Results:**

Central administration of exendin-4 in lean mice increased sympathetic outflow towards BAT and white adipose tissue (WAT), resulting in increased thermogenesis as evidenced by increased uncoupling protein 1 (UCP-1) protein levels and decreased lipid content, while the uptake of TG-derived fatty acids was increased in both BAT and WAT. Interestingly, in DIO mice, the effects on WAT were blunted, while exendin-4 still increased sympathetic outflow towards BAT and increased the uptake of plasma TG-derived fatty acids and glucose by BAT. These effects were accompanied by increased fat oxidation, lower plasma TG and glucose concentrations, and reduced body weight.

**Conclusions/interpretation:**

Collectively, our results suggest that BAT activation may be a major contributor to the glucose- and TG-lowering effects of GLP-1R agonism.

## Introduction

Glucagon-like peptide-1 (GLP-1) receptor (GLP-1R) agonists, such as exendin-4, have been used in the treatment of type 2 diabetes; they increase glucose-dependent insulin secretion, regulate gastric emptying and reduce food intake and body weight [1]. At least some of these actions are mediated through neuroendocrine mechanisms, as shown by rodent studies, consistent with the notion that GLP-1R is highly expressed in the hypothalamus. Intracerebroventricular (i.c.v.) administration of GLP-1 reduces food intake and body weight [2, 3]. Moreover, the central GLP-1R signalling system is linked to the control of peripheral glucose metabolism by inhibiting non-insulin-mediated glucose uptake by muscle and increasing insulin secretion from the pancreas [4, 5]. Previously, we have shown that subcutaneous GLP-1 treatment reduces hepatic glucose production in mice, partly through central GLP-1R signalling [6]. Hence, one of the functions of the central GLP-1R system is the modulation of the metabolic activity in peripheral organs that are crucial for the maintenance of energy homeostasis.

Brown adipose tissue (BAT) is a regulator of overall energy homeostasis by combusting triacylglycerol (TG) and glucose into heat. Enhancement of the thermogenic capacity of BAT induces weight loss through increased energy expenditure [7, 8] and lowers the plasma levels of TG and glucose. In fact, BAT activation can correct hyperlipidaemia [9] and hyperglycaemia [10] by increased uptake of TG-derived fatty acids [11] and glucose from plasma. Recently, it has been shown that activation of central GLP-1R increases thermogenesis in BAT and induces browning within white adipose tissue (WAT), and that this correlates with increased expression of genes required for thermogenesis, including uncoupling protein-1 [12, 13]. While these findings suggest an important role of the central GLP-1R system in BAT activation, the effect of central GLP-1R activation on lipid and glucose control by BAT has not yet been investigated. Furthermore, although GLP-1R agonists are implemented to treat type 2 diabetes, the responsiveness of the central GLP-1R system to activate BAT to take up TG-derived fatty acids and glucose under central insulin resistance [14] is unknown.

The aim of this study was to evaluate the potential of activating central GLP-1R by exendin-4 to facilitate clearance of lipids and glucose from the circulation by activating BAT, under both insulin-sensitive and insulin-resistant conditions in diet-induced obese (DIO) C57Bl/6J mice.

## Methods

### Animals

For all experiments, 20-week-old male C57Bl/6J mice (Charles River, Saint-Germain-Nuelles, France) were used, housed with a regular 12:12 h light/dark cycle in a temperature- and humidity-controlled environment, with free access to food and water unless noted otherwise. For diet-induced obesity, mice were fed a high-fat diet (44% energy derived from bovine fat; Hope Farms, Woerden, the Netherlands) for 12 weeks, starting at 8 weeks of age. All animal experiments were performed in accordance with the regulations of the Dutch law on animal welfare, and the Institutional Ethics Committee for Animal Procedures, Leiden University Medical Center, Leiden, the Netherlands, approved the protocol.

### Intracerebroventricular surgery and treatment

Mice were randomised based on body weight, anaesthetised and cannulas (Brain Infusion Kit 3, ALZET Cupertino, CA, USA) were stereotactically implanted into the left lateral ventricle of the brain (coordinates −0.45 mm anteroposterior, −1.00 mm lateral and 2.50 mm dorsoventral from bregma). Osmotic minipumps (Model 1004, Alzet, CA, USA) attached to the cannula via a catheter were implanted subcutaneously on the back, slightly posterior to the scapulae. The catheter connected to the osmotic minipump was filled with artificial cerebrospinal fluid (aCSF; Harvard Apparatus, Holliston, MA, USA) to delay the start of drug delivery by 2 days. The minipump assured continuous delivery of 0.75 nmol/day exendin-4 (Bachem, Weil am Rhein, Germany) dissolved in aCSF or aCSF only. The dose of exendin-4 was expected to suppress food intake. Therefore, an aCSF-receiving pair-fed control group (restricted to the same amount of food each day as consumed by the i.c.v. exendin-4-infused mice) was also included. Because of the inclusion of a pair-fed group, blinding to group assignment was not feasible. Body weight and food intake were measured daily. One lean mouse (assigned to exendin-4 group) and three obese mice (assigned to control group) were excluded from the study because of complications after surgery. After 5 days of intervention, TG and glucose clearance was determined as described below.

### Triacylglycerol and glucose clearance

Triacylglycerol-rich lipoprotein (TRL)-like particles were prepared from 100 mg of total lipid including glycerol trioleate (triolein; 70 mg), egg yolk phosphatidylcholine (22.7 mg), lysophosphatidylcholine (2.3 mg), cholesteryl oleate (3.0 mg) and cholesterol (2.0 mg), with addition of glycerol tri[^3^H]oleate ([^3^H]TO) (3.7 MBq). The emulsion was sonicated and fractionated by consecutive density gradient ultracentrifugation steps [15]. The emulsion fraction containing TRL-like particles with an average size 80 nm was isolated and mixed with 2-[1-^14^C]deoxy-d-glucose ([^14^C]DG) in a 3:1 ratio based on radioactive count. Emulsions were stored at 4°C under argon and used for in vivo kinetic experiments within 5 days following preparation.

Animals were fasted for 4 h. At *t* = 0, blood was drawn via the tail vein to determine, via enzymatic assays, basal plasma TG (Roche Molecular Biochemicals, Indianapolis, IN, USA) and glucose (Instruchemie, Delfzijl, the Netherlands). Mice then received an i.v. injection of TRL-like particles (1 mg TG) and [^14^C]DG. Blood samples were taken from the tail vein at 2, 5, 10 and 15 min after injection, and plasma ^3^H and ^14^C activities were counted. After the last blood sample, the mice were killed, perfused via the heart with ice-cold PBS and various organs were collected. Organs were dissolved overnight at 60°C in Tissue Solubilizer (Amersham Biosciences, Roosendaal, the Netherlands), and ^3^H and ^14^C activities were counted. Uptake of [^3^H]TO- and [^14^C]DG-derived radioactivity by the organs was calculated from the ^3^H and ^14^C activities in each organ and expressed as percentage of injected dose per g wet tissue weight.

### Body composition

After the treatment period, body composition (lean and fat mass) was determined in conscious DIO mice using an EchoMRI-100 (EchoMRI, Houston, TX, USA).

### Indirect calorimetry

During the treatment period of the DIO mice, oxygen uptake ($\overset{\cdot }{V}{\mathrm{O}}_2$), carbon dioxide production ($\overset{\cdot }{V}{\mathrm{CO}}_2$) and physical activity were measured in metabolic cages (LabMaster System, TSE Systems, Bad Homburg, Germany). The average respiratory exchange ratio (RER), energy expenditure and carbohydrate and fat oxidation rates were calculated from day 1 to day 4 of treatment, as described previously [16].

### Histology

Formalin-fixed paraffin-embedded interscapular BAT (iBAT) and subcutaneous WAT (sWAT) tissue sections (5 μm) were stained with haematoxylin and eosin (H&E) using standard protocols. For staining of UCP-1 and tyrosine hydroxylase (TH), sections were dewaxed, rehydrated and treated with peroxidase. Antigen retrieval was accomplished in 10 mmol/l citrate buffer (pH = 6.0). Slides were blocked with normal goat serum (UCP-1) or BSA (TH) and incubated overnight at 4°C with anti-UCP-1 antibodies (1:4,000; Ab10983; Abcam, Cambridge, UK) or anti-TH antibodies (1:2,000; Ab112; Abcam). Next, sections were incubated for 30 min with biotinylated goat α-rabbit secondary antibodies (UCP-1; 1:600; Vector Labs, Burlingame, CA, USA) or DAKO EnVision anti-rabbit antibodies (DAKO, Glostrup, Denmark). Immunostaining was amplified and visualised using the Elite ABC Nova Red kit (Vector Labs). Counterstaining was performed with Mayer’s haematoxylin (1:4). The areas occupied by intracellular lipid vacuoles, UCP-1 and TH protein were quantified using ImageJ.

### Statistical analysis

Differences between groups were determined with the Kruskal–Wallis non-parametric test. When significant differences were found, the Mann–Whitney non-parametric test was used as a post hoc test to determine differences between two independent groups. Serum decay in the clearance experiment was analysed using repeated-measurements ANOVA with Tukey’s post hoc test. A *p* value of < 0.05 was considered statistically significant. Data are presented as mean ± SD.

## Results

### Central GLP-1R activation decreases body weight and induces BAT activation in lean C57Bl/6J mice

**Fig. 1:**
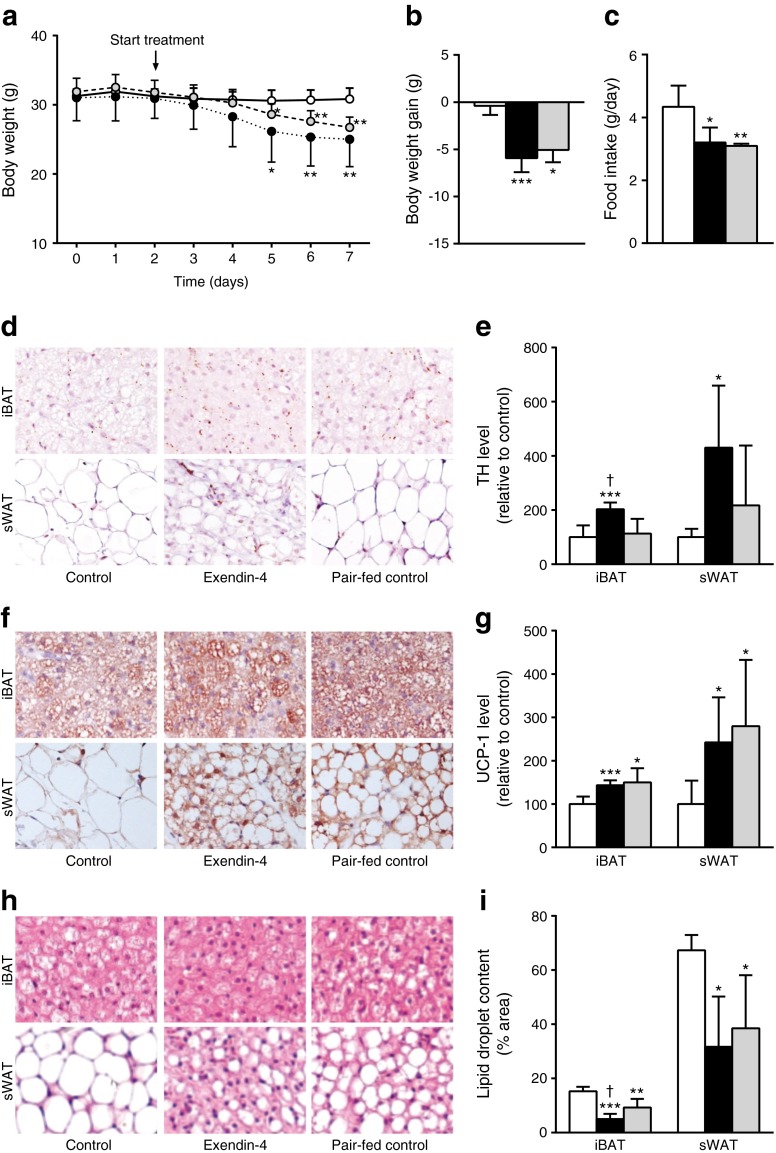
Central GLP-1R activation decreases body weight and food intake and induces activation of BAT in lean mice. Mice were treated for 5 days with i.c.v. exendin-4 (*n* = 8) or vehicle (control *n* = 9, pair-fed *n* = 6). On a daily basis, body weight (**a**, **b**) and food intake (**c**) were monitored. Samples of BAT and WAT were collected and stained for TH and UCP-1. Representative pictures and quantification of TH (**d**, **e**), UCP-1 (**f**, **g**) and lipid droplet content with H&E staining (**h**, **i**) are shown. Values are mean ± SD. *p < 0.05, **p < 0.01 and ***p < 0.001 compared with control; ^†^ *p* < 0.01 compared with pair-fed control. White bars, control; black bars, exendin-4; grey bars, pair-fed control

Intracerebroventricular infusion of exendin-4 in lean C57Bl/6J mice decreased body weight compared with controls (Fig. 1a, b), and this was accompanied by a reduced food intake (Fig. 1c), both of which are well-known effects of activation of the central GLP-1R system [3, 17]. Reduction of food intake per se, as shown by the control mice that were pair fed with the exendin-4-treated mice (Fig. 1c), resulted in a reduction in body weight compared with controls that was delayed compared with that evoked by exendin-4 (Fig. 1a).

As central GLP-1 infusion increases sympathetic outflow towards BAT and WAT and subsequently stimulates thermogenesis [12, 13, 18], we analysed the expression of TH, a marker for activity of noradrenergic (norepiphrenergic) nerve fibres [19]. Exendin-4 increased the TH content in both iBAT (+103%, *p* < 0.01) and sWAT (+331%, *p* < 0.05) compared with controls (Fig. 1d, e). This increased sympathetic nervous system (SNS) signalling was accompanied by enhanced thermogenic capacity, as exendin-4 increased UCP-1 protein content in iBAT (+44%, *p* < 0.01) and sWAT (+142%, *p* < 0.05) compared with the control group (Fig. 1f, g), indicating more active BAT as well as browning of WAT. Reduction of food intake per se evoked similar increases in UCP-1 protein content in both BAT and WAT, but it was apparently independent of sympathetic input, which suggests the involvement of other pathways. The effects of exendin-4 on UCP-1 content were accompanied by a decreased lipid droplet content in iBAT (−67%, *p* < 0.001) and sWAT (−53%, *p* < 0.05) (Fig. 1h, i), probably as activation of BAT results in combustion of intracellular lipid stores as well as browning of WAT. The lipid content in iBAT was reduced to a greater extent in exendin-4-treated mice than in food-restricted mice (−46%, *p* < 0.05), which coincided with a higher level of TH, reflecting higher SNS activity towards BAT induced by exendin-4.

### Central GLP-1R activation enhances uptake of plasma TG-derived fatty acids and glucose by BAT and browns WAT in lean C57Bl/6J mice

**Fig. 2:**
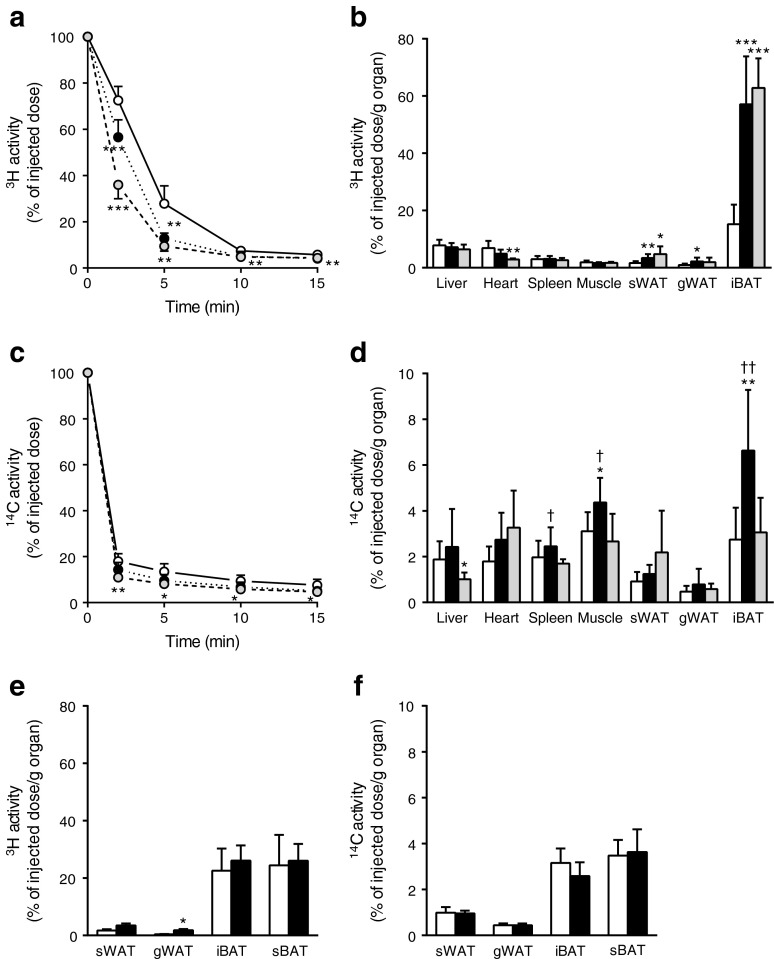
Central GLP-1R activation increases uptake of plasma TG-derived fatty acids and glucose by BAT in lean mice. Mice were treated for 5 days via i.c.v. (**a**–**d**) or i.v. (**e**–**f**) routes with exendin-4 (*n* = 8) or vehicle (control *n* = 9, pair-fed *n* = 6). After treatment, mice were injected with [^3^H]TO-labelled particles and [^14^C]DG. Plasma ^3^H activity (**a**) and ^14^C activity (**c**) were plotted relative to the injected dose. At 15 min after injection, organs were isolated and uptake of ^3^H activity (**b**, **e**) and ^14^C activity (**d**, **f**) was determined. Values are mean ± SD. *p < 0.05, **p < 0.01 and ***p < 0.001 compared with control. ^†^ *p* < 0.05 and ^††^ *p* < 0.01 compared with pair-fed control. White bars/circles, control; black bars/circles, exendin-4; grey bars/circles, pair-fed control

Subsequently, we tested our hypothesis that BAT activated during chronic GLP-1R activation is a major contributor to the plasma clearance of TG and glucose. To this end, mice were injected i.v. with [^3^H]TO-labelled TRL-like emulsion particles and [^14^C]DG. Indeed, exendin-4 accelerated the clearance of [^3^H]TO from plasma (Fig. 2a). This enhanced clearance was the result of a greatly increased uptake of [^3^H]TO-derived activity by iBAT (+276%, *p* < 0.001; Fig. 2b). In addition, exendin-4 enhanced the uptake of [^3^H]TO-derived activity by both sWAT (+111%, *p* < 0.01) and gonadal (g)WAT (+138%, *p* < 0.05) compared with the control group, corresponding to the browning observed in these WAT depots. The pair-fed animals showed similar results for [^3^H]TO kinetics as the exendin-4-treated group. Plasma clearance was increased (Fig. 2a), relating to a marked increase in the uptake of ^3^H activity by iBAT compared with control-infused animals (+314%, *p* < 0.001; Fig. 2b). In addition, the uptake by sWAT was increased (+194%, *p* < 0.05), whereas there was no significant effect on uptake by gWAT (+109%, *p* = 0.20) in pair-fed conditions.

Similar to plasma [^3^H]TO clearance, chronic infusion of exendin-4 accelerated the clearance of [^14^C]DG from plasma (Fig. 2c). This enhanced clearance from the circulation was the result of an increased uptake of [^14^C]DG by iBAT (+142%, *p* < 0.01) and skeletal muscle (+40%, *p* < 0.05) compared with controls (Fig. 2d). Interestingly, while the uptake of [^3^H]TO-derived activity by BAT in pair-fed mice was increased to a similar extent as in exendin-4 treated animals, the uptake of [^14^C]DG by iBAT was not increased in the pair-fed control mice.

To rule out that the effects of i.c.v. administered exendin-4 on the uptake of TG-derived fatty acids and glucose by BAT were caused by leakage of exendin-4 into the circulation, we next administered exendin-4 peripherally at the same dose as administered centrally. Peripheral exendin-4 treatment for 5 days delivered via subcutaneous minipumps did not enhance the uptake of [^3^H]TO-derived activity (Fig. 2e) or [^14^C]DG (Fig. 2f) by BAT. Peripheral exendin-4 only slightly increased the uptake of [^3^H]TO-derived activity by gWAT, possibly via a direct effect of GLP-1R signalling on white adipocyte formation [20].

### Central GLP-1R activation decreases body weight, plasma TG and glucose levels, and shifts combustion from carbohydrates towards fat in DIO C57Bl/6J mice

**Fig. 3:**
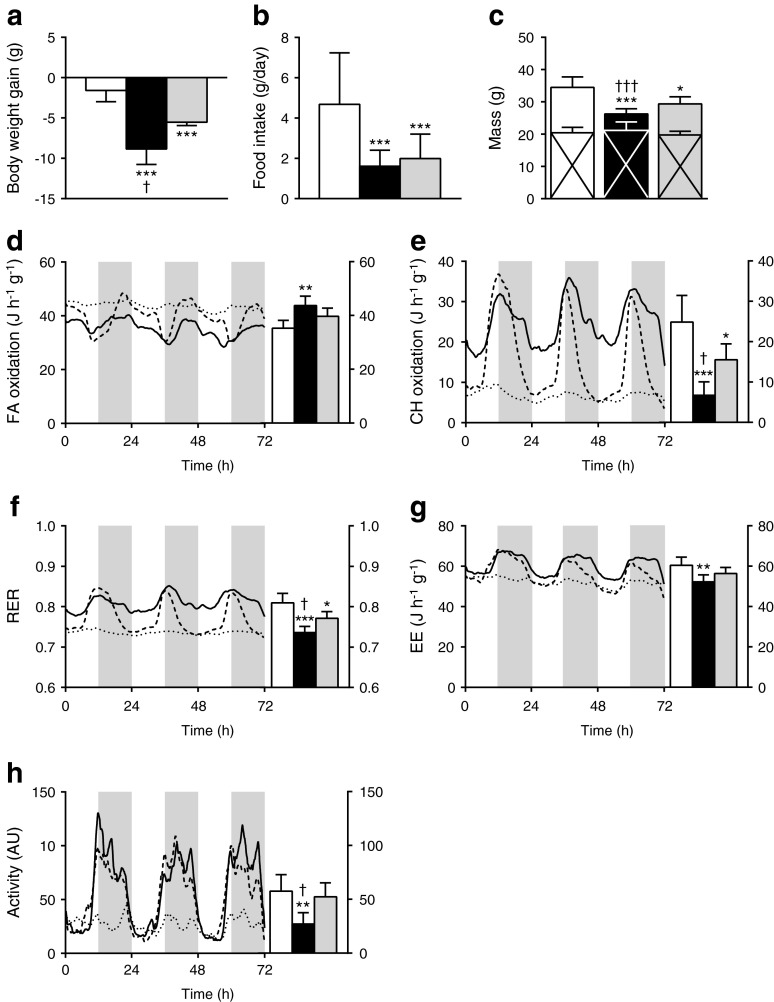
Central GLP-1R activation decreases body weight and shifts combustion from carbohydrates towards fat in DIO mice. After 12 weeks of high-fat feeding, mice were treated for 5 days with i.c.v. exendin-4 (*n* = 10) or vehicle (control *n* = 6, pair-fed *n* = 9). Body weight gain (**a**) and food intake (**b**) were determined. Lean (crossed area) and fat (no cross) mass were measured (**c**). During the treatment period, mice were housed in metabolic cages. Fatty acid oxidation (**d**), carbohydrate oxidation (**e**), RER (**f**) and energy expenditure (**g**) were calculated from O_2_ uptake and CO_2_ excretion. (**h**) Magnitude of physical activity in arbitrary units. Values are mean ± SD. *p < 0.05, **p < 0.01 and ***p < 0.001 compared with control. ^†^ *p* < 0.05 and ^†††^ *p* < 0.001 compared with pair-fed control. White bars/continuous lines, control; black bars/dotted lines, exendin-4; grey bars/dashed lines, pair-fed controls. AU, arbitrary units; CH, carbohydrate; EE, energy expenditure; FA, fatty acid

To determine whether chronic i.c.v. exendin-4 infusion still leads to an increased uptake of plasma TG-derived fatty acids and glucose via BAT when obesity and insulin resistance have developed, we next explored the effects of central GLP-1R activation after 12 weeks of high-fat feeding. In DIO mice, continuous infusion of exendin-4 for 5 days decreased body weight compared with control-infused mice (Fig. 3a), which could only partly be attributed to reduced food intake (Fig. 3b) as the reduction in body weight was greater than in pair-fed controls. Determination of body composition showed that exendin-4 and pair feeding decreased body weight because of a selective decrease in fat mass compared with controls (Fig. 3c). Furthermore, plasma TG and glucose levels were both decreased from baseline values in the exendin-4-infused mice at the end of the treatment period (Table 1). In the pair-fed mice, plasma glucose levels were reduced from baseline values, but remained significantly higher than in the exendin-4-treated mice.

**Table 1:** Central GLP-1R activation decreases plasma TG and glucose levels in DIO mice

Variable	Control	Exendin-4	Pair-fed control
TG (mmol/l)			
Start	0.7 ± 0.1	0.7 ± 0.1	0.6 ± 0.1
End	0.7 ± 0.5	0.5 ± 0.2*	0.6 ± 0.4
Glucose (mmol/l)			
Start	11.3 ± 0.8	11.6 ± 1.1	11.4 ± 1.1
End	10.6 ± 0.9	4.5 ± 1.2*** ^,^^†††^	7.4 ± 1.1***

After 12 weeks of high-fat feeding, mice were treated for 5 days with i.c.v. exendin-4 (*n* = 10) or vehicle (control *n* = 6, pair-fed *n* = 9). Blood was collected before and after treatment by tail bleeding after 4 h of fasting and plasma TG and glucose were determined

Values are mean ± SD

*p < 0.05 and ***p < 0.001 compared with baseline; ^†††^ *p* < 0.001 compared with pair-fed control

Indirect calorimetry showed that the decreased fat mass in the exendin-4-infused DIO mice was accompanied by an increased fat oxidation (Fig. 3d) at the expense of carbohydrate oxidation (Fig. 3e). This was also reflected in a decreased RER (Fig. 3f) compared with controls, suggesting a shift in nutrient combustion. Of note, exendin-4 reduced total energy expenditure (Fig. 3g) and decreased physical activity (Fig. 3h). Pair feeding also increased fat oxidation and decreased carbohydrate oxidation compared with the controls, but these effects were less pronounced than those of exendin-4. This implies that the effects of exendin-4 on energy metabolism are only partly caused by reduced food intake in DIO mice.

### Central GLP-1R activation increases BAT thermogenesis, but does not induce browning of WAT in DIO C57Bl/6J mice

**Fig. 4:**
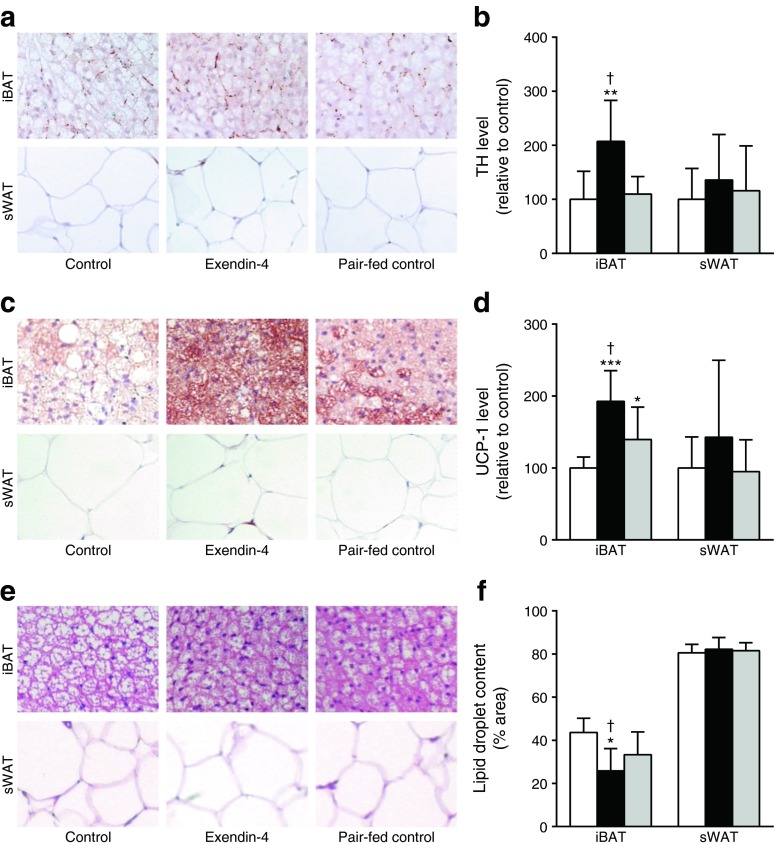
Central GLP-1R activation increases TH and UCP-1 protein levels and decreases lipid droplet content in BAT in DIO mice. After 12 weeks of high-fat feeding, mice were treated for 5 days with i.c.v. exendin-4 (*n* = 10) or vehicle (control *n* = 6, pair-fed *n* = 9). After treatment, mice were killed and BAT and WAT were collected and stained for TH and UCP-1. Staining with H&E was performed for lipid droplet content. Representative pictures and quantification of TH (**a**, **b**), UCP-1 (**c**, **d**) and lipid droplet content (**e**, **f**) are shown. Values are mean ± SD. *p < 0.05, **p < 0.01 and ***p < 0.001 compared with control. ^†^ *p* < 0.01 compared with pair-fed control. White bars, control; black bars, exendin-4; grey bars, pair-fed control

Under high-fat-fed conditions, i.c.v. exendin-4 treatment still increased SNS output towards BAT compared with controls, reflected by increased TH content (sBAT +59%, *p* < 0.05 [not shown]; iBAT +107%, *p* < 0.01, Fig. 4a, b) and increased UCP-1 protein content (sBAT +62% [not shown], *p* < 0.001; iBAT +93%, *p* < 0.01, Fig. 4c, d), indicative of more active BAT. As a consequence, lipid droplet content was decreased in the exendin-4-treated animals compared with controls (sBAT −49%, *p* < 0.05 [not shown]; iBAT −41%, *p* < 0.01, Fig. 4e, f). Pair feeding did not affect TH expression in BAT (Fig. 4a, b), slightly increased UCP-1 protein content (Fig. 4c, d) and slightly decreased lipid content (Fig. 4e, f) compared with controls. In contrast to the lean mice, infusion of exendin-4 in DIO mice did not induce browning of WAT as evidenced by unaffected TH expression, UCP-1 protein and lipid content.

### Central GLP-1R activation enhances TG and glucose clearance by BAT in DIO C57Bl/6J mice

**Fig. 5:**
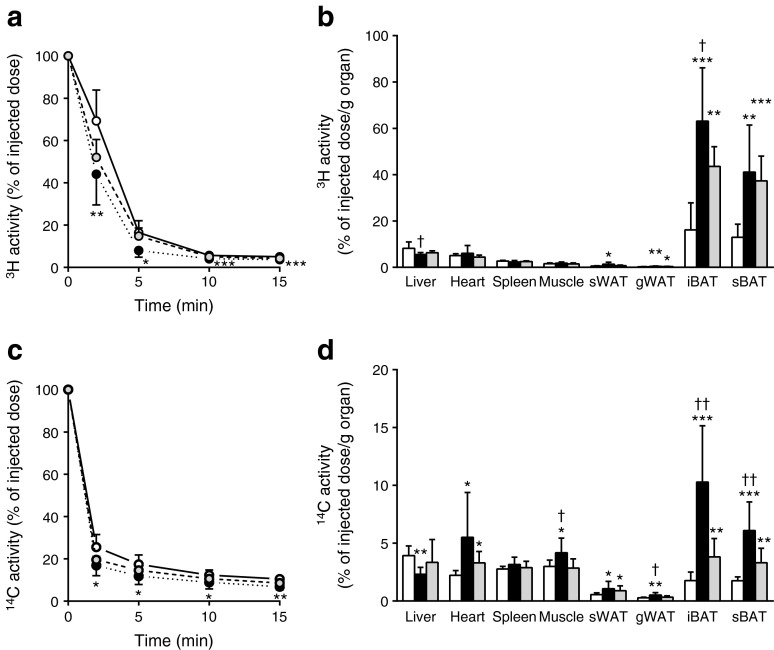
Central GLP-1R activation increases uptake of plasma TG-derived fatty acids and glucose by BAT in DIO mice. After 12 weeks of high-fat feeding, mice were treated for 5 days with i.c.v. exendin-4 (*n* = 10) or vehicle (control *n* = 6, pair-fed *n* = 9). After treatment, mice were injected with [^3^H]TO-labelled particles and [^14^C]DG. Plasma ^3^H activity (**a**) and ^14^C activity (**c**) were plotted relative to the injected dose. At 15 min after injection, organs were isolated and uptake of the ^3^H activity (**b**) and ^14^C activity (**d**) was determined. Values are mean ± SD. *p < 0.05, **p < 0.01 and ***p < 0.001 compared with control. ^†^ *p* < 0.05 and ^††^ *p* < 0.01 compared with pair-fed control. White bars, control; black bars, exendin-4; grey bars, pair-fed control

Similar to lean mice, chronic i.c.v. infusion of exendin-4 in DIO mice accelerated the plasma clearance of [^3^H]TO (Fig. 5a) and [^14^C]DG (Fig. 5c). This was accompanied by a selectively increased uptake of [^3^H]TO-derived activity and [^14^C]DG by iBAT (+291%, *p* < 0.001 and +482%, *p* < 0.001, respectively) and sBAT (+217%, *p* < 0.01 and +247%, *p* < 0.001, respectively) (Fig. 5b, d). Despite the lack of evidence for browning of WAT, exendin-4 enhanced the uptake of both [^3^H]TO-derived activity and [^14^C]DG by both sWAT (+146%, *p* < 0.05 and +93%, *p* < 0.05) and gWAT (+69%, *p* < 0.01 and +82%, *p* < 0.01). To some extent, the pair-fed animals showed similar results on [^3^H]TO and [^14^C]DG kinetics as the exendin-4 treated mice. Plasma clearance was increased (Fig. 5a, c), associated with a marked increase in uptake of [^3^H]TO-derived activity and [^14^C]DG by iBAT (+170%, *p* < 0.01 and +482%, *p* < 0.001, respectively) and sBAT (+188%, *p* < 0.001 and +247%, *p* < 0.001, respectively) compared with control-infused animals (Fig. 5b, d). Interestingly, the uptake of [^3^H]TO-derived activity by iBAT and the uptake of [^14^C]DG by iBAT and sBAT were significantly lower compared with the exendin-4-treated mice (−31%, *p* < 0.05, −63%, *p* < 0.01 and −43%, *p* < 0.01, respectively), indicating that exendin-4 exerts its effects partly independent of lowering food intake.

## Discussion

In the present study we show that central GLP-1R activation by exendin-4 increases the plasma clearance of TG and glucose in both lean and DIO C57Bl/6J mice via increased uptake of TG-derived fatty acids and glucose by BAT, accompanied by activation of BAT and browning of WAT.

First, we showed that continuous central infusion of the GLP-1R analogue exendin-4 (5 days, 0.75 nmol/day) evokes the well-known effect of reducing food intake and body weight [3, 17]. In addition, central administration of exendin-4 increased SNS activity towards BAT and WAT, as evidenced by increased TH and UCP-1 protein content and lowered lipid content. This corroborates previous findings demonstrating the essential role of the SNS in BAT and WAT activation by central GLP-1R signalling [12, 18]. Exendin-4 also caused a robustly accelerated clearance of plasma TG and glucose, which likely contributed to reduced plasma TG and glucose concentrations observed in DIO mice. Strikingly, the uptake of TG-derived activity, presumably [^3^H]oleate liberated by lipoprotein lipase (LPL) [11], was not only increased by BAT but also by WAT, together with the increase in UCP-1 content indicative of so-called browning. Although activation of BAT and WAT are likely involved in the observed reduction of plasma TG and glucose by central exendin-4, further studies are warranted to investigate the quantitative contribution of GLP-1R signalling towards BAT and WAT to the overall metabolic improvements on GLP-1R agonism.

It has been reported that central GLP-1 infusion reduces the lipid content of WAT in lean mice but not DIO mice [18], suggesting that some resistance to the actions of central GLP-1 is induced in DIO mice. Clearly, this effect is not desirable for a drug to treat obesity. Therefore, we explored the effects of chronic central exendin-4 infusion after 12 weeks of high-fat feeding, sufficient to induce obesity and insulin resistance in this mouse model [21, 22]. Our results show that under these conditions, chronic central GLP-1R signalling still improves the clearance of plasma TG and glucose via a robustly increased uptake by BAT. However, central GLP-1R agonism did not alter UCP-1 protein content or lipid droplet content in WAT in DIO mice, consistent with previous findings by Nogueiras et al [18]. It is interesting to speculate on why, during obesity, WAT is not susceptible to browning on exendin-4 treatment. Previous studies reported a decreased sensitivity of white adipocytes to adrenergic stimulation in obese individuals [23]. However, in our study we also describe the absence of exendin-4-induced TH expression in WAT in DIO mice. It is possible that different brain areas modulate specific BAT and WAT functions. While multiple tissues are simultaneously sympathetically stimulated during cold exposure, there are many examples of treatments that result in differential sympathetic outflow to various types of tissues (e.g. WAT vs BAT) and even within a type of tissue (e.g. different WAT pads) [24]. The GLP-1R is widely expressed through the hypothalamus [25], and exerts effects via different nuclei. For example, GLP-1R signalling in the arcuate nucleus regulates glucose metabolism, while it modulates feeding via the paraventricular nucleus [5]. Altogether, it is likely that GLP-1R signalling and subsequent sympathetic outflow differ during obesity and lean conditions.

In apparent contrast to the notion that increased BAT activity is generally correlated with an enhanced total energy expenditure [26], central GLP-1R activation in fact reduced total energy expenditure, an effect we have observed before during chronic peripheral exendin-4 treatment [27]. Possibly, the reduction in energy intake is compensated by a relative reduction in energy expenditure. In addition, it appears that chronic central GLP-1R activation results in a shift from using carbohydrates to fatty acids as an energy source, which is consistent with previous studies that showed that chronic i.c.v. GLP-1 treatment decreased the respiratory quotient, indicative of a higher level of fat oxidation by BAT [18, 28]. The exendin-4-induced decrease in physical activity, resulting from a diminished food-seeking behaviour [29], is less likely to contribute to the change in total energy expenditure [30].

From a clinical perspective, the possibility that BAT activity in humans may be amenable to pharmacological manipulation by GLP-1R agonism to control insulin sensitivity and body weight is attractive but, as yet, undemonstrated. Drugs targeting the GLP-1R system are already widely prescribed for their incretin properties to treat type 2 diabetes, but they may be useful in a wider context related to energy balance. The resting energy expenditure of obese individuals with type 2 diabetes increases with 1 year of treatment with a combination of metformin and exenatide or liraglutide [13]. It is tempting to speculate that BAT may be activated in these patients. Increasing our knowledge about the mechanism of action of exendin-4 may add to the (further) development of peptidomimetics in our battle against obesity and type 2 diabetes.

In conclusion, our results show that chronic central infusion of exendin-4 increases SNS output to enhance BAT activity in both lean and DIO C57Bl/6J mice. Via highly active BAT, the plasma clearance of TG and glucose is accelerated and body fat content is decreased, which, together with reduced food intake, leads to a decrease in body weight. Therefore, we suggest that GLP-1R agonists via BAT activation reduce both hyperlipidaemia and hyperglycaemia, and possibly even atherosclerosis [31], in addition to the effects of BAT activation on obesity.

## Abbreviations

[^14^C]DG: 2-[1-^14^C]Deoxy-d-glucose
[^3^H]TO: Glycerol tri[9,10(n)-^3^H]oleate
aCSF: Artificial cerebrospinal fluid
BAT: Brown adipose tissue
DIO: Diet-induced obese
GLP-1: Glucagon-like peptide-1
GLP-1R: Glucagon-like peptide-1 receptor
gWAT: Gonadal white adipose tissue
iBAT: Interscapular brown adipose tissue
i.c.v.: Intracerebroventricular
RER: Respiratory exchange ratio
sBAT: Subscapular brown adipose tissue
SNS: Sympathetic nervous system
sWAT: Subcutaneous white adipose tissue
TG: Triacylglycerol
TH: Tyrosine hydroxylase
TRL: Triacylglycerol-rich lipoprotein
UCP-1: Uncoupling protein-1
WAT: White adipose tissue
